# Pullout Behavior of Bundled Aramid Fiber in Fiber-Reinforced Cementitious Composite

**DOI:** 10.3390/ma13071746

**Published:** 2020-04-09

**Authors:** Toshiyuki Kanakubo, Saki Echizen, Jin Wang, Yu Mu

**Affiliations:** 1 Division of Engineering Mechanics and Energy, University of Tsukuba, Ibaraki 305-8573, Japan; 2 Department of Engineering Mechanics and Energy, University of Tsukuba, Ibaraki 305-8573, Japan; s1820881@s.tsukuba.ac.jp (S.E.); wangjin20115@yahoo.co.jp (J.W.); s1530211@u.tsukuba.ac.jp (Y.M.)

**Keywords:** bundled fiber, embedded length, inclined angle, pullout load, bridging law

## Abstract

The tensile performance of fiber-reinforced cementitious composite (FRCC) after first matrix cracking is characterized by a tensile stress–crack width relationship called the bridging law. The bridging law can be obtained by an integral calculus of forces carried by individual bridging fibers considering the effect of the fiber inclination angle. The main objective of this study is to investigate experimentally and evaluate the pullout behavior of a single aramid fiber, which is made with a bundling of original yarns of aramid fiber. The bundled aramid fiber has a nonsmooth surface, and it is expected to have good bond performance with the matrix. The test variables in the pullout test are the thickness of the matrix and the inclined angle of the fiber. From the test results, the pullout load–slip curves showed that the load increases lineally until maximum load, after which it decreases gradually. The maximum pullout load and slip at the maximum load increase as the embedded length of the fiber becomes larger. The pullout load–crack width relationship is modeled by a bilinear model, and the bridging law is calculated. The calculated result shows good agreement with the experimental curves obtained by the uniaxial tension test of aramid–FRCC.

## 1. Introduction

In the past several decades, a number of types of fiber-reinforced cementitious composites (FRCCs) such as engineered cementitious composite (ECC) [1], strain hardening cement composite (SHCC) [2], and ductile fiber-reinforced cementitious composite (DFRCC) [3] have been introduced and studied by many researchers. ECC and SHCC show strain hardening and multiple fine cracking behavior under uniaxial tension. FRCCs showing a deflection hardening and multiple crack under bending condition are defined as DFRCCs. The pullout behavior of a single fiber from a cementitious matrix is one of the keys for discussing the tensile characteristics of FRCCs [4]. The tensile characteristics of FRCCs after the first cracking of matrix are featured by the bridging performance of fibers in which fibers are pulled out from the matrix. In the case of straight steel fibers, the effect of the inclined angle and yield strength have been investigated by Naaman and Shah [5] and Leung and Shapiro [6]. In addition, the rupture of fibers creates a brittle fracture of FRCCs in the case of polymer fibers such as polyethylene (PE), polyvinyl alcohol (PVA), and polypropylene (PP) [7].

To perform the experimental studies to investigate the pullout behavior of a single fiber is considered to be not easy, because thin fibers with small diameters less than 100 μm have been utilized in the above-mentioned FRCCs [8]. Several researchers have studied the pullout behavior of a single polymer fiber. Kanda and Li conducted the pullout test for a single PVA fiber with a diameter of 14 μm [9]. They concluded that PVA fiber has high chemical bond and frictional bond strength. They also pointed out that the apparent rupture strength of fiber in matrix is lower than the tensile strength measured by the tensile test of a fiber. Redon et al. also performed the pullout test for PVA fibers with diameters of 44 μm and 700 μm and reported that the small diameter PVA fiber ruptured due to high bond strength [10]. Before those experiments, Wang et al. carried out the pullout test for a nylon and PP fiber with a diameter of 508 μm [11] and showed the differences of pullout behavior between two fibers. Li et al. also conducted the pullout test for a nylon and PE fiber with the same diameter [12]. The snubbing effect in which the pullout strength increases as the inclined angle of the fiber becomes larger was investigated. Kiyota et al. compared the pullout behavior of aramid, PVA, and PE fibers through pullout test results [13]. The tested fibers were a single yarn of aramid, PVA, and PE fibers with the diameters of 12 μm, 37 μm, and 12 μm, respectively. It can be considered that the pullout behavior of a single fiber differs by each fiber type and dimension. The subbing effect and the apparent rupture strength should be also confirmed for each type of fiber.

The tensile performance of FRCCs after the first cracking of matrix are characterized by a tensile stress–crack width relationship called the bridging law. The bridging law can be obtained by an integral calculus of forces carried by individual bridging fibers, considering the probability density function for fiber inclination angle and fiber centroidal location [14]. The authors have also studied the bridging law for PVA–FRCC proposing a new probability density function based on the results of visualization simulation using a water glass solution [15]. The pullout load versus crack width relationship for a single PVA fiber has been expressed by a trilinear model in this study. The bridging law has also been modeled by some characteristic points given as the function of fiber orientation intensity [16]. The authors have also conducted experimental research studies for structural elements using FRCCs such as coupling beams [17] and beam–column joints [18,19]. For example, Li has introduced a scheme from basic materials design theory to practical commercial applications for ECC technologies [20]. Thus, it is important to investigate and evaluate the pullout behavior of a single fiber to sufficiently make the most of the advantage of FRCCs.

Aramid fiber is known as one of the polymeric fibers that have high tensile strength. Aramid fiber has been used for strengthening of concrete structures by the external bonding of a fiber sheet. A few research studies can be found concerning FRCC mixed with aramid discrete fibers [21,22]. A commercially provided aramid single fiber has a small diameter of 12 μm, and a high bond strength between cementitious matrix cannot be expected [13]. In the case of PVA fiber, it has been considered that the alcohol group in a PVA molecule brings good bond in a matrix. In other types of polymer fibers, the smooth surface of a single fiber does not generate large bond resistance. The contrivances to make good bond performance such as a hooked end, twisted, deformed surface, etc. are generally applied for steel fibers [23].

The main objective of this study is to investigate experimentally and evaluate the pullout behavior of a single aramid fiber, which is made with a bundling of original yarns of aramid fiber. The bundled aramid fiber shows a nonsmooth surface, and it is expected to have good bond performance with the cementitious matrix by mechanical resistance-like deformed steel fiber. This study aims to clarify the pullout characteristics of the bundled aramid fiber from the viewpoint of the effect of the embedded length and inclined angle of fiber. At first, the pullout test of a single bundled fiber is conducted. After that, calculation of the bridging law using a single fiber pullout model is conducted and the calculation result is compared with the uniaxial tension test results of a FRCC.

## 2. Experimental Program

### 2.1. Tested Aramid Fiber

**Figure 1:**
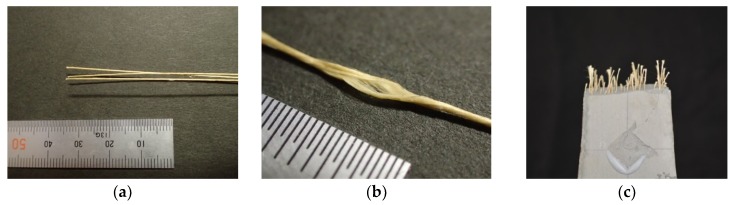
Aramid fiber used in this study: (**a**) Bundled fiber for pullout test; (**b**) Condition of bundling of yarns; (**c**) Example of chopped fiber in fiber-reinforced cementitious composite (FRCC).

The aramid fiber used in this study is a bundled fiber with a nominal diameter of 500 μm. The used fiber is shown in Figure 1. The original yarns of aramid fibers with a nominal diameter of 12 μm (Technora, TEIJIN [24]) are twisted to form a thick single fiber and sized not to unravel in the FRCC. The tensile strength and elastic modulus of the original yarn is 3432 MPa and 73 GPa, respectively, according to the manufacturer test results. A continuous single fiber before cutting is prepared for the pullout test. Chopped fibers with a length of 30 mm are utilized for mixing the FRCC.

### 2.2. Specimen for Pullout Test

**Figure 2:**
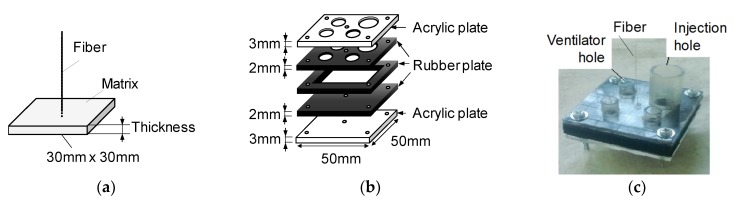
Pullout specimen: (**a**) Dimensions of pullout specimen; (**b**) Constitute of mold; (**c**) Example of mold.

**Figure 3:**
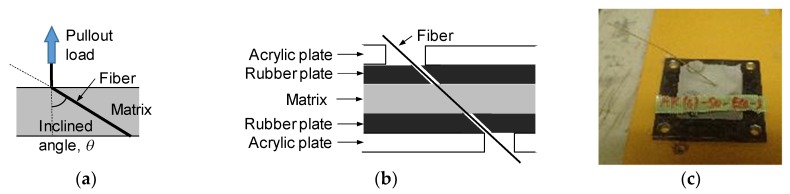
Pullout specimen with inclined fiber: (**a**) Definition of inclined angle; (**b**) Constitute of mold and fixing of fiber; (**c**) Example of specimen with inclined fiber.

Figure 2 shows the details and mold for the pullout test specimen. The pullout specimen is the thin plate made of cementitious matrix in which a single fiber is embedded at the center of the plate. The dimension of the plane section is a 30 mm × 30 mm square, and the thickness of the plate is one of the test variables. The mold consists of two acrylic plates and three rubber plates. A total of five plates are fixed by bolts not to cause visible deformation of the rubber plates. A single fiber is positioned by the holes of the upper and lower rubber plates. A cementitious matrix is poured from the injection hole and the ventilator holes function not to make air voids. The thickness of the specimen is varied by changing the thickness of the middle rubber plate. The other test variable is the inclined angle of the fiber. Figure 3 shows the details of the specimen with the inclined fiber. The angle is set by the position of the hole in the lower rubber plate. The specimen is set to the loading machine via a steel plate that is adhered on the bottom surface of the specimen.

The test variables are the thickness of the matrix and the inclined angle of the fiber. The thickness is varied as 2, 4, 6, 8, and 12 mm. Since the target aramid fiber used in FRCC has a 30 mm length, thicknesses smaller than the half-length of the fiber were chosen. The inclined angle is set to 0, 15, 30, 45, and 60 degrees. The embedded length of the fiber is obtained by the specimen thickness divided by cos*θ*, where *θ* expresses the inclined angle.

The list of the specimens is shown in Table 1. The fabrication of specimens is grouped into four series by mixing batches (A, B, C, and D). Each test series includes no-inclined angle specimens (0 degree) and inclined angles of 15, 30, 45, or 60 degree specimens. Five specimens for each angle and thickness were fabricated. In order to check the difference of mixing batches of the matrix mixture, no-inclined angle specimens were tested for each series. The total number of specimens is 200, including 100 no-inclined angle specimens.

Table 2 shows the mixture proportion of the cementitious matrix. The unit weight of water was 380 kg/m^3^, and fine sand under 0.2 mm diameter was used. The mixture proportion is designed for FRCC with self-consolidating properties.

### 2.3. Loading Method

**Figure 4:**
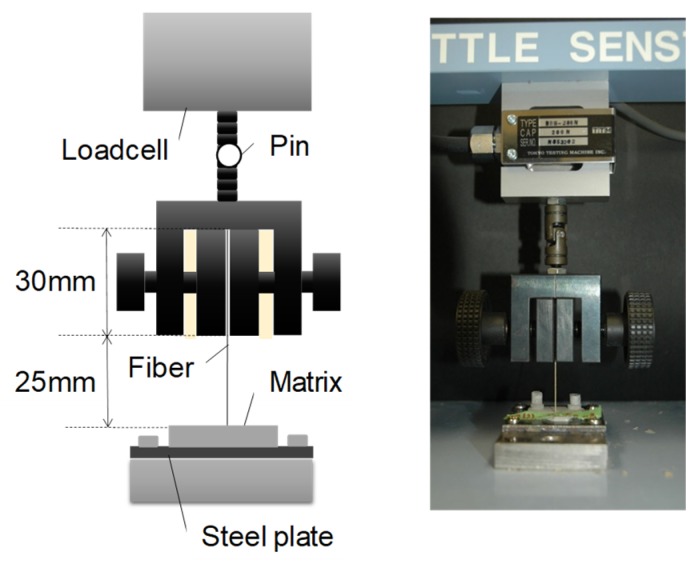
Loading method.

A monotonic pullout load was applied using an electronic system universal testing machine with the capacity of 200 N (LSC-02/30-2, Tokyo Koki Testing Machine Co., Ltd., Tokyo, Japan), as shown in Figure 4. The specimen was fixed via an adhered steel plate, and the embedded fiber was clamped directly by a chucking jig. The length of fiber out of the matrix was 55 mm, and the gripping length of the fiber is equal to that of the jig. The head speed was set to 1 mm per minute. The pullout load and head displacement were recorded.

## 3. Experimental Result

### 3.1. Failure Progress

**Figure 5:**
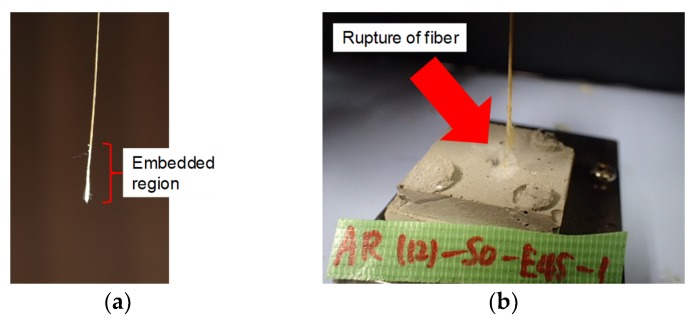
Examples of tested fiber and specimen after loading: (**a**) Pulled out fiber; (**b**) Rupture of fiber.

**Figure 6:**
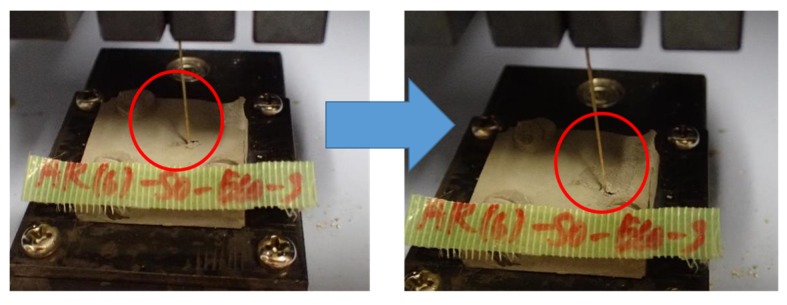
Example of the specimen with peeling of matrix.

In most of the specimens, the fiber was pulled out from the matrix without observing a clear rupture of the fiber. As shown in Figure 5a, the surface of the fiber embedded in the matrix was damaged, and some unraveled original yarns were observed. In some specimens with inclined angles of 30, 45, and 60 degrees, a clear rupture of fiber or peeling of the matrix around the embedded fiber was observed. As shown in Figure 5b, fiber ruptured inside the matrix. Figure 6 shows an example of the specimen with any peeling of the matrix. Specimens of 12 mm thickness with an inclined angle of 60 degrees peeled off from the steel plate, so the suitable data could not be obtained.

### 3.2. Pullout Load–Slip Curve

**Figure 7:**
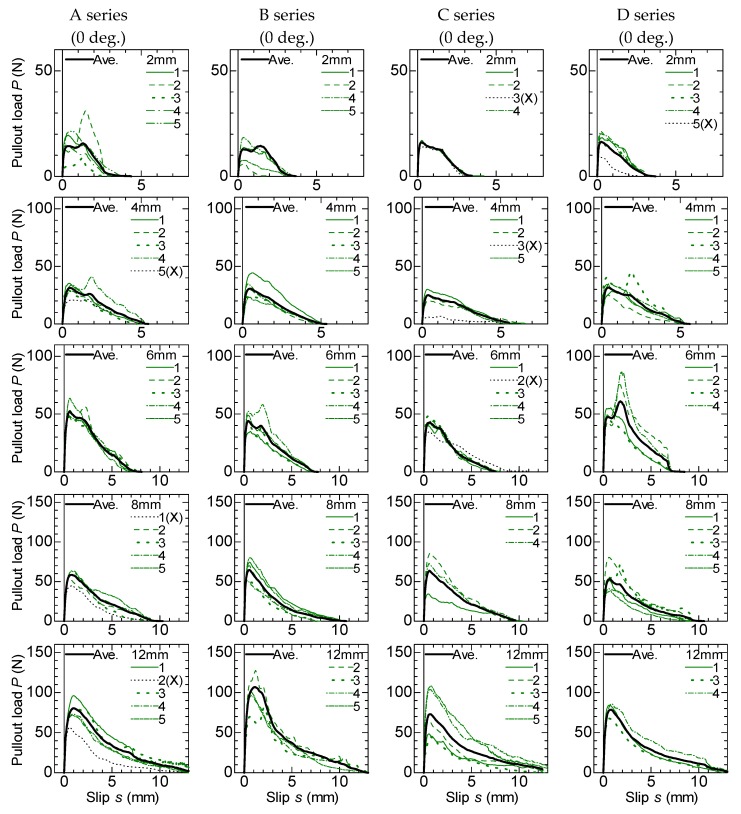
Pullout load–slip curve (no-inclined angle specimen).

Pullout load–slip curves of no-angle specimens are shown in Figure 7. The graphs are listed by specimen series in the columns and thicknesses in the rows. There are no differences among the test variables except for the mixing batches in the specimens, which are listed in the same row. The number in each graph indicates the identification of each specimen in the same test variables. Although five specimens for each test series were fabricated, some specimens could not be loaded because of the breakage of the matrix when specimens were remolded. The slip is calculated from the measured displacement of the loading head subtracting the elongation of the fiber outside the matrix based on the tension test results for a single fiber. The averaged curves are calculated in each series of specimens to compare the curves between the different series of specimens. Since dispersion among each series of specimens is observed, the curve that shows that the maximum load smaller than 90 percentiles is excluded in the calculation of the averaged curve. The averaged curves are shown by thick lines in Figure 7, and the excluded specimens for the calculation are indicated by “X”.

As shown in Figure 7, the curves generally show two stages, i.e., the load increases lineally until the maximum load, and then it decreases gradually. The pullout load becomes almost zero when the slip reaches the thickness of specimen, which is equal to the embedded length of fiber in the case of no-angle specimens. The maximum load generally increases as the thickness also increases. It is considered that these observed phenomena indicate that the bond resistance of the bundled aramid fiber is due to the constant bond stress along the embedded fiber, which is similar to a friction mechanism. The large differences of the averaged curves between the different series of specimens with same thickness are not observed.

**Figure 8:**
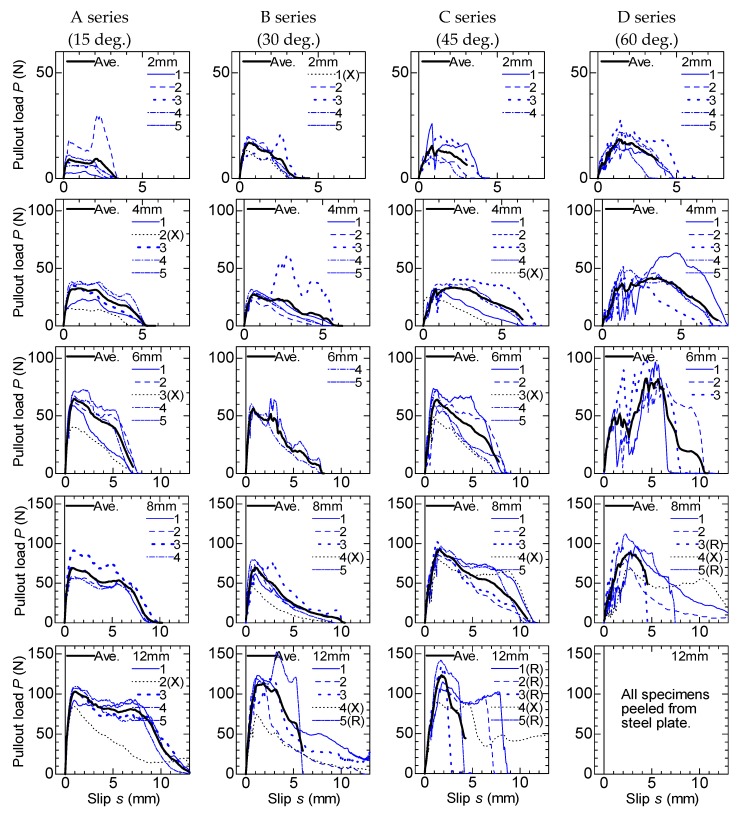
Pullout load–slip curve (inclined angle specimen).

Pullout load–slip curves of specimens with the inclined fiber are shown in Figure 8. The graphs are listed by specimen series (inclined angle) in the columns and the thicknesses are listed in the rows. The averaged curves are shown by thick lines similarly to those in Figure 7. The “R” after the specimen identification number indicates the specimen in which clear fiber rupture was observed.

As shown in Figure 8, the load increases lineally and decreases gradually in the case of thin thickness and small inclined angle specimens, similarly to the case in which there are no-angle specimens. After the first peak, some specimens show a decreasing and re-increasing of the load at a large slip, which is because of the peeling of the matrix. The maximum load generally increases as the thickness and inclined angle also increase. Fiber rupture was observed in the specimens that showed larger pullout load.

### 3.3. Maximum Pullout Load

**Figure 9:**
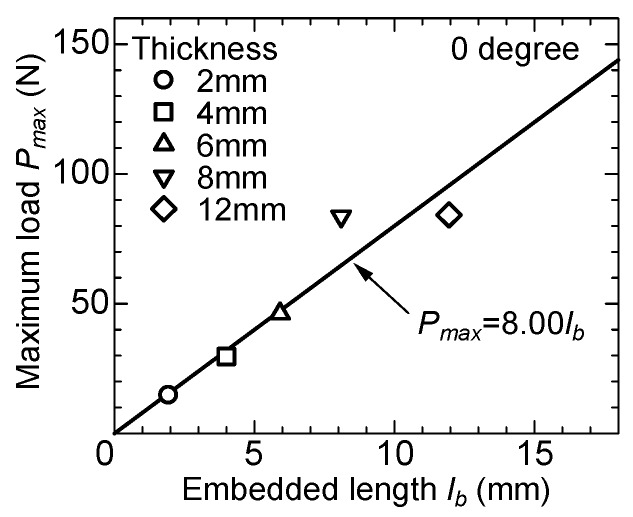
Maximum load versus embedded length (no-inclined angle).

The experimental results show that the maximum load generally increases as the specimen thickness becomes larger. In addition, a larger inclined angle also increases the maximum load. Figure 9 shows the relationship between the maximum pullout load, *P_max_*, and the embedded length, *l_b_*, of the fiber in the no-angle specimens. The maximum pullout load is the average value of the maximum loads in the averaged curves shown in Figure 7 for each thickness. The embedded length is the average of specimen thicknesses, which were measured by a caliper. The plots are positioned in an almost proportional relationship; a formula in Figure 9 is obtained by the least square method.

**Figure 10:**
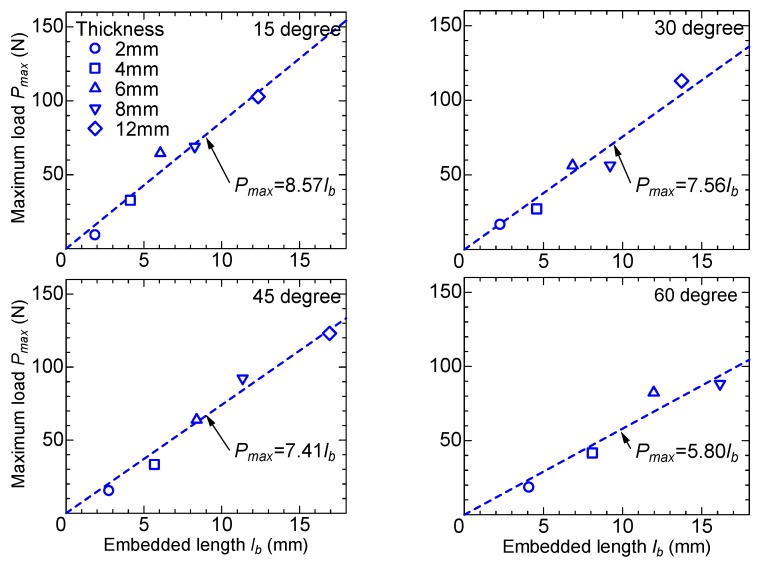
Maximum load versus embedded length (inclined angle).

Similarly, Figure 10 shows the relationship between the maximum pullout load and the embedded length of fiber in the inclined angle specimens. The embedded length is obtained by the measured thickness divided by cos*θ*, where *θ* is the inclined angle. For the inclined angle specimens, the maximum pullout loads are also expressed by proportional relations with the embedded lengths. The formulas shown in Figure 10 are obtained by the least square method.

**Figure 11:**
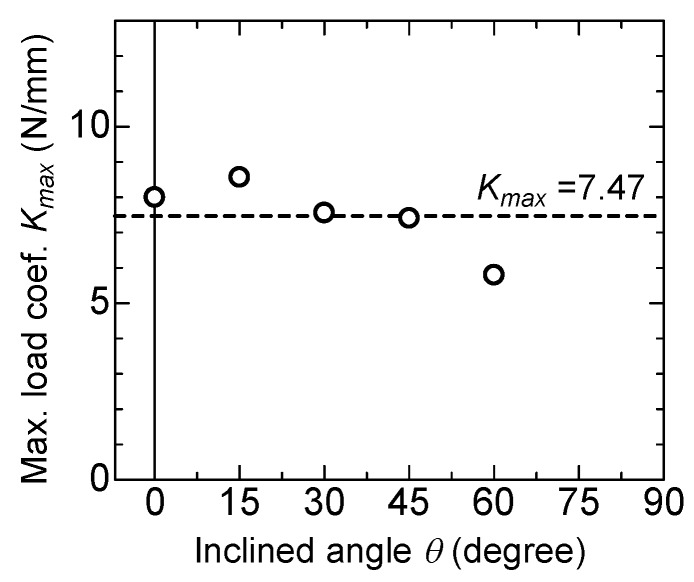
Maximum load coefficient versus inclined angle.

Figure 11 compares the proportional coefficients for the maximum load, *K_max_*, obtained by each inclined angle. It is considered that the snubbing effect confirmed in the viewpoint of the pullout load is mainly due to the increasing of the embedded length in the case of inclined fiber. So, the maximum pullout load, *P_max_*, can be expressed by Equation (1), as the average of the proportional coefficients.

(1) $$P_{\max}=7.47\cdot l_{b}$$

### 3.4. Apparent Rupture Strength of Fiber

Kanda and Li showed that the apparent rupture strength of polymer fiber in a matrix is lower than the tensile strength measured by the tensile test of the fiber [9]. The reason given is that the fiber surface is damaged by the matrix at the pullout position and embedded region.

**Figure 12:**
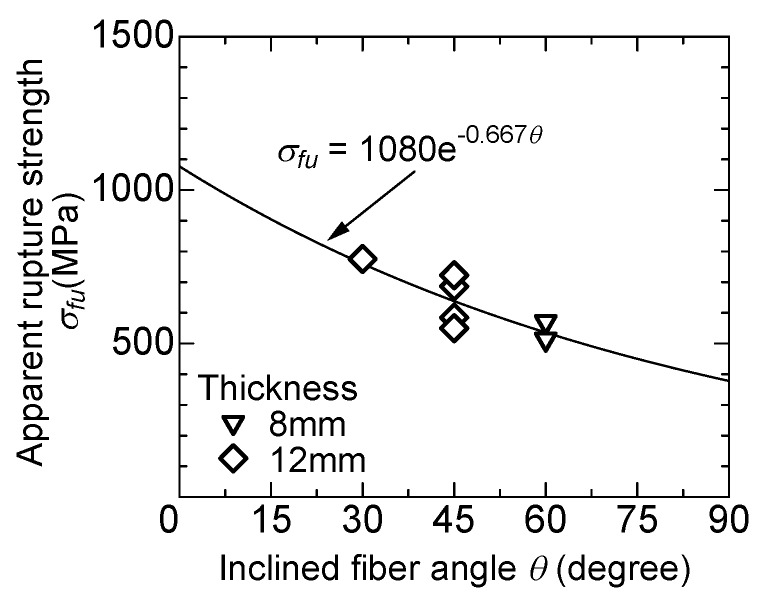
Apparent rupture strength of fiber.

Figure 12 shows the relationship between the inclined angle of the fiber and the rupture strength. The rupture strength is obtained from the maximum load divided by the cross-sectional area of the fiber in the case of the specimens that showed clear rupture of fiber (indicated by “R” in Figure 8). The rupture strength reduces as the inclined angle becomes larger, which is similar to the results of the previous study [9].

## 4. Single Fiber Pullout Model and Calculation of Bridging Law

### 4.1. Bilinear Model for Pullout Load–Slip Curve

**Figure 13:**
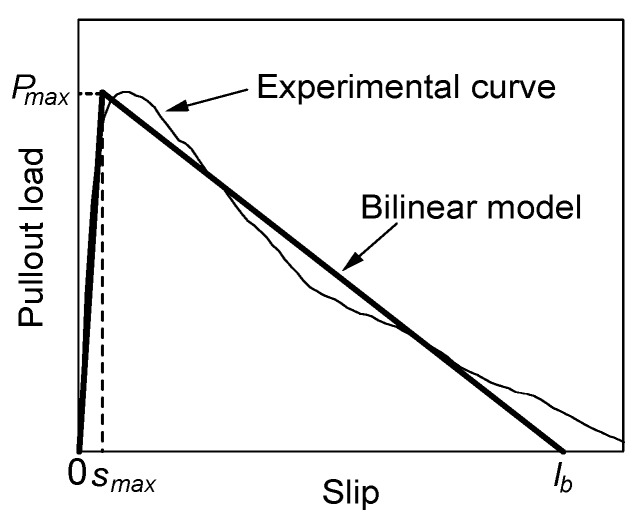
Bilinear model for pullout load–slip curve.

Based on the test results, the pullout load–slip curve is modeled by a bilinear model, as shown in Figure 13, where *P_max_* expresses the maximum pullout load and *s_max_* expresses the slip at the maximum load. The maximum pullout load can be given by Equation (1) as the average of the test results. The pullout load becomes zero at *s* = *l_b_*, which expresses the embedded length of the fiber.

### 4.2. Slip at Maximum Load

**Figure 14:**
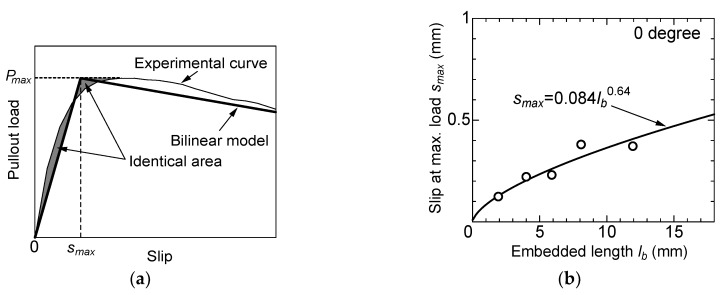
Methodology of determining the slip at the maximum load: (**a**) First line of the bilinear model with an equivalent complementary energy; (**b**) Relationship between slip at the maximum load and the embedded length of the fiber.

In order to obtain good agreements between the experimental curves and bilinear model, slip at the maximum pullout load is calculated from the averaged experimental curve to have an equivalent complementary energy until the maximum load. Figure 14a shows a sketch of the bilinear model and methodology of determining the first line of the model. The first line is determined to show the same area surrounded by the experimental curve and the first line of the model. Figure 14b shows the relationship between slip at the maximum load and the embedded length of fiber in no-angle specimens. The slip at the maximum load increases as the embedded length becomes larger. The power function gives a good fitting result, as shown in the figure. So, the slip at the maximum pullout load, *s_max_*, of the bilinear model is expressed by Equation (2).

(2) $$s_{\max}=0.084\cdot l_{b}{}^{0.64}$$

### 4.3. Calculation Method of Bridging Law

The bridging law, i.e., the tensile stress–crack width relationship, is calculated similarly to that in the previous study [15]. The bridging law can be obtained by the summation of forces carried by individual bridging fibers considering the probability density function for the fiber inclination angle and the fiber centroidal location as given by Equation (3).

(3) $$\begin{array}{ll} \sigma_{bridge} & =\frac{P_{bridge}}{A_{m}}\\ & =\frac{V_{f}}{A_{f}}\cdot \sum_{h}\sum_{j}\sum_{i}P_{ij}(w,\psi )\cdot p_{xy}(\theta_{i})\cdot p_{zx}(\phi_{j})\cdot p_{x}(y_{h},z_{h})\cdot \Delta \theta \cdot \Delta \phi \cdot (\Delta y\cdot \Delta z) \end{array}$$

where,

*σ_bridge_* = tensile stress,

*P_bridge_* = bridging force (= total of pullout load),

*A_m_* = cross-sectional area of matrix,

*V_f_* = fiber volume fraction,

*A_f_* = cross-sectional area of a single fiber,

*P*(*w*,*ψ*) = pullout load of a single fiber,

*p_xy_*, *p_zx_* = probability density function for fiber inclination angle,

*p_x_* = probability density function for fiber centroidal location,

*ψ* = fiber inclination angle to *x*-axis (= max{*θ*, *ϕ*}),

*θ* = angle between *x*-axis and projected line of the fiber to *x*-*y* plane,

*ϕ* = angle between *x*-axis and projected line of the fiber to *z*-*x* plane,

*w* = crack width.

The elliptic distribution is adopted for the probability density function for fiber inclination angle [15]. The fiber orientation varies by the value of orientation intensity, *k* (ratio of the two radii of elliptic function) and principal angle, *θ_r_* (argument of one radius of elliptic function). The random orientation is given by *k* = 1. When the value of *k* is larger than 1, fibers tend to orient toward *θ_r_*. When the value of *k* is smaller than 1, fibers tend to orient perpendicular to *θ_r_*. The probability density function for fiber centroidal location is set to be constant. This means that the fibers are randomly distributed along the longitudinal direction of the specimen.

*P*(*w*,*ψ*) expresses the pullout load of a single fiber, and it should be the model given by crack width, *w*. The crack width is considered to be the summation of slips from both sides of the embedded fiber across the crack. Similar to the previous study [15], the crack width at the maximum load is assumed to be 1.5 times the slip, because the slip of the fiber from the long embedded side does not reach the maximum pullout load. So, the crack width at the maximum pullout load, *w_max_*, is given by Equation (4).

(4) $$w_{\max}=1.5\cdot s_{\max}=0.13\cdot l_{b}{}^{0.64}$$

The rupture of the fiber is also considered in the calculation of the bridging law. Once the tensile stress of the fiber by the pullout load exceeds the apparent rupture strength shown in Figure 12, the pullout load becomes zero.

### 4.4. Comparison of Calculation Result with Tension Test Result

**Figure 15:**
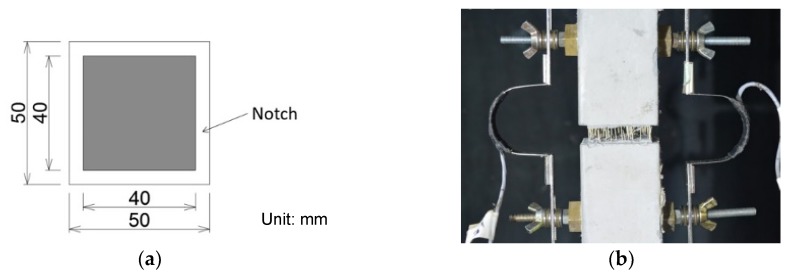
Uniaxial tension test specimen: (**a**) Dimensions of cross-section; (**b**) Specimen at loading.

A uniaxial tension test for aramid-FRCC has been performed by the authors in reference [19]. The shape of the tension test specimen is dog-bone type, and the cross-section is a 50 mm × 50 mm square with 5 mm deep notches. Figure 15 shows the dimensions of the specimen and the loading situation. The details of the uniaxial tension test can be found in the reference. The used aramid fiber and the mixture proportion of the matrix are precisely the same as in this study.

**Figure 16:**
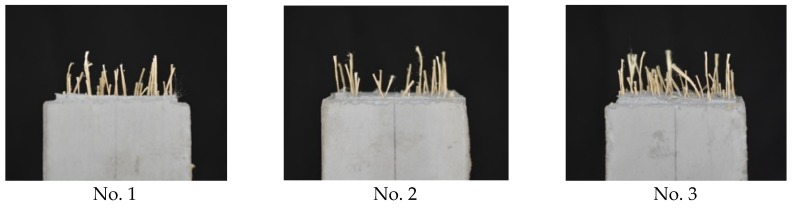
Uniaxial tension test specimen after loading (bottom side).

**Figure 17:**
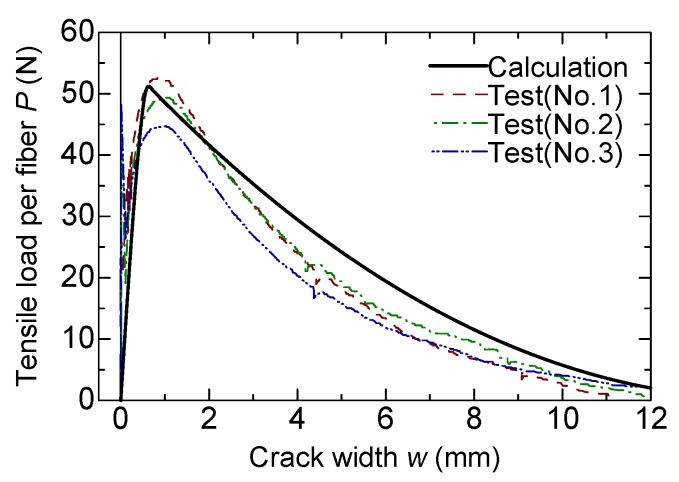
Comparison of bridging law.

The tension test specimens after loading are shown in Figure 16, and the test results are listed in Table 3. Since the observed maximum loads showed dispersion among three specimens, the numbers of fibers that appeared from the fracture surface were counted after loading. As seen in Table 3, the specimen with a larger number of fibers showed a higher maximum load. Figure 17 shows the tensile load versus crack width curves. The tensile load is divided by the total number of fibers of each specimen to express the average tensile force carried by the individual fiber.

The calculation results of the bridging law are also shown in Table 3 and Figure 17. The calculation is carried out using the bilinear model for pullout load–crack width relation described in Section 4.1, considering the inclined fiber angle and rupture of the fiber. The orientation intensity, *k*, in the elliptic distribution for the probability density function of the fiber inclination angle is set to 1.5 and 6 for two planes parallel to the axial direction, which is the same as in the previous study [15]. The principal angle, *θ_r_*, is also set to zero. The tensile load (=*P_bridge_*) is divided by the theoretical fiber number across the crack, which can be calculated by *V_f_ A_m_*/*A_f_ η_f_*, where *η_f_* is the fiber effectiveness [15] at the crack width of zero. In the case of the orientation intensities of 1.5 and 6, the fiber effectiveness is 0.544. The parameters for the calculation are summarized in Table 4.

As seen in Figure 17, the calculated bridging law shows good agreement with the experimental curves. From Table 3, the calculated tensile strength per fiber is from 0.97 to 1.14 times that of the experiment. Some differences of the shape of curves around the maximum and post-peak branch are considered to be due to the bilinear modeling of a single fiber pullout behavior.

## 5. Conclusions

Based on the results of this experimental program and calculation of the bridging law, the following conclusions are drawn:

1. In the pullout test of a single bundled aramid fiber, the fiber was pulled out from the matrix without observing clear rupture in most of the test variable cases. In some specimens with fiber inclined angles of 30, 45, and 60 degrees and a large embedded fiber length, clear rupture of the fiber or peeling of the matrix around the embedded fiber was observed.
2. Pullout load–slip curves showed that the load increases lineally until the maximum load is reached, after which it decreases gradually. The pullout load becomes almost zero when the slip reaches the embedded length of the fiber.
3. The maximum pullout load has a proportional relation with the embedded length of the fiber. It is considered that the bond resistance of the bundled aramid fiber is due to constant bond stress along the embedded fiber-like friction mechanism. A clear tendency between the proportional coefficients and inclined fiber angle is not observed. The slip at the maximum load increases as the embedded length of the fiber becomes larger. The relation between them is expressed by the power function.
4. The pullout load–crack width relationship was modeled by a bilinear model based on the results of the pullout test. The bridging law, i.e., tensile stress–crack width relationship was calculated using the model. The calculated result shows good agreement with the experimental curves obtained by the uniaxial tension test of aramid–FRCC.

## Figures and Tables

**Table 1:** Specimen list.

Test Series (Mixture Batch)	Inclined Angle (Degree)	Thickness (mm)	Number of Specimens	Total Number of Specimens
A series	0 *	2 4 6 8 12	5 for each variable	25
	15			25
B series	0 *			25
	30			25
C series	0 *			25
	45			25
D series	0 *			25
	60			25

*: There is no difference of test variables except for the mixing batches (casting date).

**Table 2:** Mixture proportion.

Water by Binder Ratio	Sand by Binder Ratio	Unit Weight (kg/m^3^)			
		Water	Cement	Fly Ash	Sand
0.39	0.50	380	678	291	484
Cement: High early strength Portland cement Fly ash: Type II of Japanese Industrial Standard (JIS A 6202) Sand: Size under 0.2 mm High-range water-reducing admixture: Binder × 0.6%					

**Table 3:** Uniaxial tension test results.

Specimen		Maximum Load (kN)	Number of Fibers			Tensile Strength per Fiber (N)
			Up Side	Bottom Side	Total	
Uniaxial tension test	No.1	5.01	49	46	95	52.7
	No.2	3.75	39	37	76	49.3
	No.3	6.40	76	67	143	44.8
Calculation		4.54	88.7 ^1^			51.2

^1^ Theoretical value (=$V_{f}\cdot A_{m}/A_{f}\cdot \eta_{f}$, $\eta_{f}$: fiber effectiveness [15]).

**Table 4:** Parameters for the calculation of the bridging law.

Parameter		Input
Cross-sectional area of a single fiber, *A_f_* (mm^2^)		0.196
Length of fiber, *l_f_* (mm)		30
Apparent rupture strength of fiber, *σ_fu_* (MPa)		$\sigma_{fu}=1080\cdot e^{-0.667\psi }$
Bilinear model	Maximum pullout load, *P_max_* (N)	$P_{\max}=7.47\cdot l_{b}$
	Crack width at *P_max_*, *w_max_* (mm)	$w_{\max}=0.13\cdot l_{b}{}^{0.64}$
Elliptic distribution	Orientation intensity for *x*-*y* plane, *k_xy_*	1.5
	Orientation intensity for *z*-*x* plane, *k_zx_*	6
	Principal orientation angle, *θ_r_* (deg.)	0
Notation: *ψ* = fiber inclination angle to *x*-axis (rad.) *l_b_* = embedded length of fiber (mm)		
